# Core Matrisome Protein Signature During Periodontal Ligament Maturation From Pre-occlusal Eruption to Occlusal Function

**DOI:** 10.3389/fphys.2020.00174

**Published:** 2020-03-05

**Authors:** Balazs Jozsef Denes, Aouatef Ait-Lounis, Bernhard Wehrle-Haller, Stavros Kiliaridis

**Affiliations:** ^1^Department of Orthodontics, Clinique Universitaire de Médecine Dentaire, University of Geneva, Geneva, Switzerland; ^2^Department of Cell Physiology and Metabolism, Centre Médical Universitaire, University of Geneva, Geneva, Switzerland

**Keywords:** maturation, post-emergent, tooth eruption, occlusal forces, extracellular matrix

## Abstract

The pre-occlusal eruption brings the molars into functional occlusion and initiates tensional strains during mastication. We hypothesized that upon establishment of occlusal contact, the periodontal ligament (PDL) undergoes cell and extracellular matrix maturation to adapt to this mechanical function. The PDL of 12 Wistar male rats were laser microdissected to observe the proteomic changes between stages of pre-occlusal eruption, initial occlusal contact and 1-week after occlusion. The proteome was screened by mass spectrometry and confirmed by immunofluorescence. The PDL underwent maturation upon establishment of occlusion. Downregulation of alpha-fetoprotein stem cell marker and protein synthesis markers indicate cell differentiation. Upregulated proteins were components of the extracellular matrix (ECM) and were characterized with the matrisome project database. In particular, periostin, a major protein of the PDL, was induced following occlusal contact and localized around collagen α-1 (III) bundles. This co-localization coincided with organization of collagen fibers in direction of the occlusal forces. Establishment of occlusion coincides with cellular differentiation and the maturation of the PDL. Co-localization of periostin and collagen with subsequent fiber organization may help counteract tensional forces and reinforce the ECM structure. This may be a key mechanism of the PDL to adapt to occlusal forces and maintain structural integrity.

## Introduction

The pre-occlusal eruption is the process that brings the tooth bud from its bony crypt into occlusion with an antagonist tooth, where it initiates its masticatory function. During this process, the fibers of the periodontal ligament (PDL) rearrange in order to acquire the tensional resistance to sustain heavy occlusal forces. This maturation also implies that the mechanical resistance of the extracellular matrix (ECM), as well as their link to ligament-embedded fibroblasts increases. While the non-cellular components of the PDL are collagens, proteoglycans and glycoproteins, they are linked to fibroblast through different types of cell adhesion molecules (CAMs) enabling anchorage to the ECM as well as migration and signaling (Alberts et al., 2014). CAMs are classified into four principal categories, immunoglobulin superfamily, cadherins, integrins, and selectins (Lodish et al., 2004), of which integrins are the principal cell-to-matrix adhesion receptors. Integrins dynamically connect ECM fibers with the contractile cytoskeleton via force-sensitive intracellular adapter proteins, such as talin and vinculin. While these intracellular adapters enable cells to sense tensional forces and allows cell adaption to their environment (Shyy and Chien, 1997; Chiquet et al., 2003), it is less clear how the ECM is reinforced due to increasing tensional forces, or how the ECM could rapidly acquire new integrin ligands to resist cell detachment in tensional stressed ligaments or tissues.

The main collagens of the PDL are collagen type I and III (Beertsen et al., 1997) with smaller portions of type V, VI (Becker et al., 1991), and XII (Karimbux and Nishimura, 1995). Collagens of the PDL form a complex network linking the tooth cementum to the alveolar bone walls and enable high loading of the tooth without damage to the supporting structures. Proteoglycans are composed of a protein core that has multiple branches of glycosaminoglycans (GAGs) attached (Bartold, 1987). They are considered to be the filler component in the ECM and can form large complexes with other proteoglycans and collagens. Proteoglycans contribute to maintaining the structure of the tissue and the ECM and protect the integrity in the face of heavy loading, for example in intervertebral disks (Roughley, 2006), in tendons (Screen et al., 2006), and in the PDL (Kurylo et al., 2016). Beyond their mechanical function, they also modulate growth factors (Zhang et al., 1995; Häkkinen et al., 2000; Kizawa et al., 2005; Yamada et al., 2008; Awata et al., 2015; Yu et al., 2018) and regulate ECM composition and structure (Matheson et al., 2005; Chen and Birk, 2013; Kurylo et al., 2016). Gylcoproteins are proteins with covalently attached N-linked glycans. They serve as extracellular or transmembrane proteins and intervene in cell-to-cell and cell-to-matrix communication. They aid in ECM assembly, cell adhesion and growth factor signaling (Hynes and Naba, 2012). The most studied glycoprotein of the PDL is periostin. It was first characterized in the PDL (Horiuchi et al., 1999), but since has been shown to be expressed in many fibrous tissues outside of the craniofacial area (Gillan et al., 2002). Other glycoproteins of the PDL include undulin and fibronectin (Zhang et al., 1993), S100 proteins (Sato et al., 1988, 1989) and Annexins (Sun et al., 2006; Wu et al., 2009). A recent effort to organize the components of the ECM has led to the creation of the matrisome database^1^, which classifies the ECM proteome (matrisome) into 2 divisions, core matrisome and matrisome-associated proteins. Each division is subdivided into three categories: collagens, ECM glycoproteins and proteoglycans (core matrisome) and ECM regulators, ECM-affiliated and secreted factors (matrisome-associated) (Naba et al., 2016).

Tooth movement has been shown to induce remodeling of the PDL through simultaneous synthesis and degradation of collagen by fibroblasts (Ten Cate et al., 1976), but little is known about the changes occurring during tooth eruption. During the transition from pre-occlusal to functional eruption the tooth reaches initial occlusal contact and eruption slows down (Denes et al., 2018). The aim of this study was to characterize the changes in matrisomal proteins of the PDL during the transition phase from the pre-occlusal to the functional phase of tooth eruption.

## Materials and Methods

### Animals

All procedures were authorized by the ethical committee of the Canton of Geneva under license number GE/72/15. Twelve male rats of *Rattus norvegicus* species and *Wistar* strain were used in this study. They were born in-house from dams acquired from Janvier Labs, France. The twelve male rats were housed in the conventional area of the Animal Facility of the University of Geneva, together with their dam until weaning on day 21 and from then on four per cage.

The animals were killed by CO_2_ at three time points, according to the rat molar eruption stages: daily *in vivo* micro-CT imaging was used to identify the pre-occlusal eruption (P18), initial occlusal contact (P21) and 1-week after occlusion (P28) with a technique previously described (Denes et al., 2018) (Supplementary Figure S1). The incisors were extracted, the mandibles were dissected and the immediately frozen with PrestoChill (Milestone^®^) in cryo-embedding at −40°C and subsequently stored at −80°C.

### Cryosectioning

For cryostat sectioning, the CryoJane Tape-Transfer was used (Leica^®^). Before sectioning, PET-membrane slides were pre-coated overnight as per supplier instructions with solution A of the kit. The alcohol baths, PET-membrane slides, CryoJane Tape and embedded mandible were placed inside of the cryostat 20 min prior to sectioning. Before cutting each section, 2.5 μl of solution B were applied to the membrane and spread to a uniform layer with a dental microbrush. The blocks were sectioned at 10 μm thickness in the cryostat at −20°C and transferred to polyethylene terephthalate (PET) membrane (Leica^®^ n°11505190) slides with CryoJane Tape and polymerized with UV light (360 nm) to fix the frozen section to the membrane, after which the tape was removed. The slides were dehydrated in successive baths of 70, 95, 100% EtOH inside the cryostat. Slides were transferred to LMD microscope on dry ice and in slide holders containing silica gel beads to ensure section preservation.

### Laser Capture Microdissection (LCM)

**FIGURE 1:**
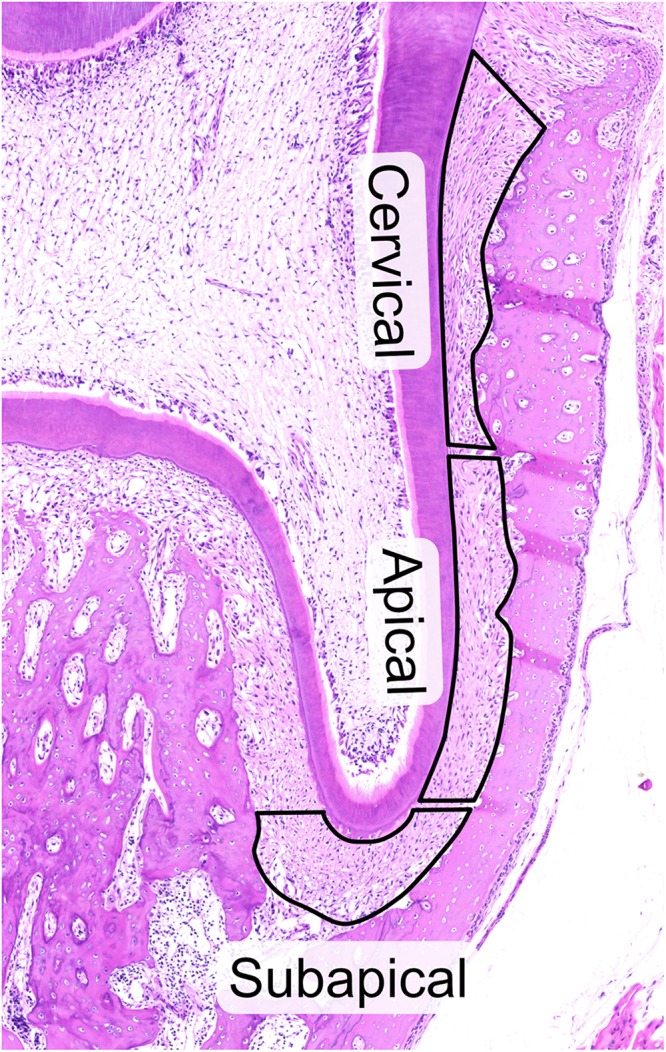
Hematoxylin and eosin staining of 28-day old rat molar showing the three regions of the periodontal ligament, cervical, apical and subapical that were laser-capture microdissected. All cuts were made inside of the PDL to avoid contamination by cementum and bone and to facilitate cutting. Attention was taken to avoid pulpal cells at subapical region and PDL invaginations in the alveolar bone. The upper limit of the cervical region was defined by the upper most border of the alveolar bone to avoid contamination of PDL by gingival tissue.

The slides were laser microdissected with Leica LMD 6500 at 50x magnification into 0.5ml Axygen tubes. The PDL of the second root of the first mandibular molar was divided into three regions of interest, cervical, apical and subapical (Figure 1). The cervical area was defined as the cervical half of the PDL between the cemento-enamel junction and the tip of the root and the apical region as the apical portion of the same region. Both areas were equally long. The subapical region was defined as the PDL underneath the apex of the root, between the two root tips. Tissue collection was done in dry tubes, which were placed in −80°C awaiting proteomic extraction. Microdissected surface areas were standardized to approximately 10^6^ μm^2^.

### Sample Preparation for Proteomic Mass Spectrometry

A total of 48 frozen samples was analyzed by the Proteomic Core Facility of the University of Geneva according to the experimental design (Supplementary Figure S1). The alveolar bone samples were not used in this study. Samples were thawed for 30 min at room temperature and a fast-spin was performed. The cap of each tube was then rinsed with 50 μl of Triethylammonium bicarbonate (TEAB) 0.1 M, 20% acetonitrile (ACN) solution and samples were spun down. Samples were dried under speed-vacuum and then resuspended in 25 μl of TEAB 0.1M, Rapigest 0.1% (Waters). Volume of resuspension was adapted for each sample in order to have the same area concentration (i.e., the sample with the smallest area was set as the reference and resuspended in exactly 25 μl). Samples were then heated to 100°C for 5 min. and lysis was performed by sonication (6 × 30 s) at 70% amplitude and 0.5 pulse. Samples were kept for 30 s on ice between each cycle of sonication. Samples were centrifuged at 13’500 rpm for 30 s. Each sample volume was adjusted to 25 μl, and then protein reduction was performed by addition of 0.5 μl of Tris(2-carboxyethyl)phosphine hydrochloride (TCEP) 50 mM in water for 1 h at 60°C. Cysteine alkylation was performed by addition of 0.5 μl of iodoacetamide 200 mM in water for 1 h at room temperature (RT) and in the dark. Overnight digestion was performed at 37°C with 1 μl of freshly prepared trypsin (Promega; 0.2 μg/μl in TEAB 0.1 M). Samples were spun down and 3.3 μl of each of the 48 LCM samples were taken to create a control pool (mix1) and the eight additional LCM samples were also pooled together to create a second control (mix2). Labels from six TMT-10plex isobaric label reagent set (Thermo Scientific) were each dissolved in 18.5 μl of ACN (i.e., 60 label reagents in total) and added to the different samples as well as the split mix1 and mix2 controls (Supplementary Figure S2).

TMT labeling was performed for 1 h at RT and stopped by addition of 2 μl of hydroxylamine 5% for 15 min. at room temperature. Labeled samples of each TMT10 experiment were pooled together, acidified with 50 μl of Trifluoroacetic acid (TFA) and let react for 45 min. at 37°C in order to cleave the Rapigest. Samples were then centrifuged 20 min. at 13’300 rpm and supernatants were transferred to new tubes. Samples were dried under speed-vacuum, desalted with a C18 microspin column (Harvard Apparatus, Holliston, MA, United States) according to manufacturer’s instructions, dried again under speed-vacuum and stored at −20°C.

### Electrospray Ionization Liquid-Chromatography-Mass Spectrometry/Mass Spectrometry (ESI-LC-MSMS)

Samples were diluted in 40 μl of loading buffer (5% CH3CN, 0.1% FA) and 4 μl were injected on the column. LC-ESI-MS/MS was performed on an Orbitrap Fusion Lumos Tribrid mass spectrometer (Thermo Fisher Scientific) equipped with an Easy nLC1200 liquid chromatography system (Thermo Fisher Scientific). Peptides were trapped on an Acclaim pepmap100, C18, 3 μm, 75 μm × 20 mm nano trap-column (Thermo Fisher Scientific) and separated on a 75 μm × 500 mm, C18, 2 μm, 100 Å Easy-Spray column (Thermo Fisher Scientific). The analytical separation was run for 125 min using a gradient of H2O/FA 99.9%/0.1% (solvent A) and CH3CN/FA 80%/0.1% (solvent B). The gradient was run as follows: 0–2 min 92% A and 8% B, then to 72% A and 28% B in 105min, then to 58% A and 42% B in 20 min and finally to 5% A and 95% B in 10 min with a stay for 23 min at this composition. Flow rate was of 250 nL/min. Data-dependent analysis (DDA) was performed with MS1 full scan at a resolution of 120’000 FWHM followed by as many subsequent MS2 scans on selected precursors as possible within 3 s maximum cycle time. MS1 was performed in the Orbitrap with an AGC target of 4 × 105, a maximum injection time of 50 ms and a scan range from 375 to 1500 m/z. MS2 was performed in the Orbitrap using higher-energy collisional dissociation HCD at 38% NCE. Isolation windows was at 0.7 u with an AGC target of 5 × 104 and a maximum injection time of 86 ms. A dynamic exclusion of parent ions of 60 s. with 10 ppm mass tolerance was applied.

### Database Search and Statistical Analysis

Raw data were processed using Proteome Discoverer (PD) 2.2 software (Thermo Fisher). Spectrum was extracted and searched against the Rattus norvegicus reference proteome database (Uniprot, release 2018-09, 31562 entries) combined with an in-house database of common contaminant using Mascot (Matrix Science, London, United Kingdom; version 2.5.1). Trypsin was selected as the enzyme, with one potential missed cleavage. Precursor ion tolerance was set to 10 ppm and fragment ion tolerance to 0.02 Da. Carbamidomethyl of cysteine (+57.021) as well as TMT10plex (+229.163) on lysine residues and on peptide N-termini were specified as fixed modification. Oxidation of methionine (+15.995) was set as variable modifications. The search results were validated with percolator for a *q*-value set at 0.01. PSM and peptides were filtered with a false discovery rate (FDR) of 1%, and then grouped to proteins with again a FDR of 1% and using only peptides with high confidence level. Both unique and razor peptides were used for quantitation and protein and peptides abundances values were based on S/N values of reporter ions. The abundances were normalized on “Total Peptide Amount” and then scaled with “On Controls Average” (i.e., using mix1 abundances as reference). All the protein ratio was calculated from the medians of the summed abundances of replicate groups and associated *p*-values were calculated with an ANOVA test based on individual protein or peptides. Protein families and interactions of the PDL proteome for the three regions and the three time points was analyzed with STRING database v11.0^2^ with organism Rattus Norvegicus and MCL clustering with inflation parameter 3. In STRING networks, proteins are represented by nodes and the interaction between proteins is given as an edge. Details of STRING analyses can be consulted online with the permalink included in the figure legend. The mass spectrometry proteomics data have been deposited to the ProteomeXchange Consortium via the PRIDE partner repository with the dataset identifier PXD013379 and 10.6019/PXD01337.

### Immunofluorescence

Immunofluorescent staining was performed to characterize matrisome protein expression and confirm proteomic profiling results. Frozen sections were used at 3 μm thickness. CryoJane tape was employed to transfer the section to the slide, which were then fixed in paraformaldehyde 4% and washed 2 × 5 min in PBST (phosphate buffered saline with 1% triton X-100). Cryosections where stained with Annexin-A1 (Anxa1, Abcam ab214486, 1:500), Asporin (Aspn, Abcam ab58741, 1:500), Biglycan (Bgn, Abcam ab49701, 1:50), Collagen A1 (III) (Col3a1, Abcam ab6310, 1:200), Lumican (Lum, Abcam ab168348, 1:200), Periostin (Postn, Abcam ab14041, 1:200), Protein S100-A6 (s100a6, Abcam ab244301, 1:50), and Protein S100-A10 (s100a10, Abcam ab187201, 1:50). Primary antibodies were incubated for 2 h, followed by secondary antibodies Alexa Fluor 488 (goat anti-mouse, Invitrogen A21121), Alexa Fluor 488 (goat anti-rabbit, Invitrogen A11008) and Alexa Fluor 555 (goat anti-rabbit, Invitrogen A21422) during 1 h. Stained samples were mounted with Vectashield^®^ (Vector Laboratories H-1500). Imaging was performed with the LSM 700 confocal laser scanning microscope (Zeiss) at 1 Airy Unit (AU) with wavelengths 416, 485, and 555 nm at 200x magnification.

## Results

### The Matrisome of the PDL Is Rich in Collagens, Glycoproteins and Proteoglycans in the Cervical and Apical Areas

Based upon a previous study (Takimoto et al., 2015), the PDL was divided into three regions to compare their function and role in the eruption process (Figure 1). To characterize the PDL proteome, the detected amount of proteins was averaged for the three age groups to give the general proteome signature of each region within the PDL. We performed the first proteome wide characterization of the PDL by searching the TMT10plex^TM^ proteomic analysis results for members of the matrisome database (Table 1A). The ratio of core matrisome and matrisome-associated proteins was higher at a ratio of 1:1 compared to the complete matrisome database ratio at 1:4. The difference was mostly due to a higher proportion of collagens (2.75-fold) and of proteoglycans (2.22-fold) as well as glycoproteins and ECM regulators (1.5-fold). We detected 14 different collagen types, of which type II, IV, XI, XIV, XVI are novel discoveries in the PDL. It is worth noting that cystatins (protease inhibitors) and anti-coagulation proteins (Serpin gene family) represent an important fraction of the ECM regulating matrisome category. Furthermore, annexins make up half of the ECM-affiliated proteins and S100 proteins almost the totality of secreted factors.

**TABLE 1:** Proteomic analysis by Tandem Mass Tag 10-plex^TM^ (TMT10plex^TM^, Thermo Fischer) mass spectrometry with ages averaged (P18, P21, P28) to reflect overall proteome of PDL.

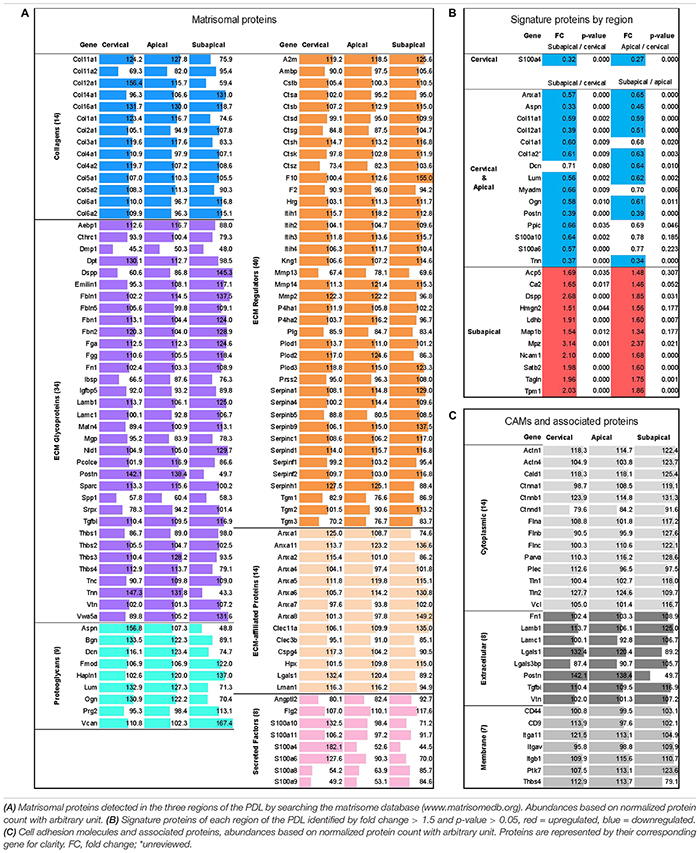

Signature proteins of each region were identified by looking at the upregulated proteins of each region or grouped regions (i.e., Cervical and Apical group) compared to the other regions. The three age groups were averaged. Out of a total of 1568 master proteins detected, 27 showed differential expression between the three regions others (Table 1B). The cervical and apical regions were remarkably similar and displayed only one differentially expressed protein, the S100A4, which may be a marker for the cervical PDL. The subapical region expression pattern was markedly different from the cervical and apical regions. Furthermore, the fold change between subapical/cervical had a tendency to be consistently greater than that of subapical/apical indicating a progressive change in PDL composition. We concluded that the signature proteins of the cervical and apical regions are matrisomal proteins, such as collagens, periostin, tenascin N, and annexin A1.

We further examined the proteome of the PDL by searching for CAMs and cell-to-matrix adhesion associated proteins (Table 1C) that ensure the link between the cells and ECM. Catenins, actinins and filamins as well as talin 1 and 2 and vinculin are well described cytoskeleton associated proteins known to link actin (Brakebusch and Fässler, 2003). These proteins bind to transmembrane proteins, of which we detected several integrin isoforms (αV, α11, β1), antigens CD9 and CD44 and thrombospondin-4. Integrins can connect directly to ECM collagens or through extracellular adhesion proteins such as fibronectin, periostin, laminins and vitronectin. Of particular interest, periostin and thrombospondin-4 have particularly high abundances in the cervical and apical areas compared to the subapical (FC = 3.6, *p*-value < 0.001 and FC = 3.3, *p*-value < 0.001 respectively), suggesting that they may play a particular role in these regions.

### The Cervical and Apical Protein Expression Changes Following Occlusal Contact

TMT10 analysis between age groups P28 (1-week post-occlusion) and P18 (pre-occlusal eruption) revealed 40 differentially expressed proteins, of which 11 were upregulated and 28 downregulated (Table 2A). The majority of the differential expression was seen in the cervical region, while the apical region showed similar changes, although with fewer proteins reaching statistical significance. The portion of the PDL closest to the occlusal forces undergoes the most marked changes possibly because the force is unequally distributed along the PDL, concentrating in the cervical area (Eskitascioglu et al., 2004; Sevimay et al., 2005). In contrast to the cervical and apical regions, the subapical region showed very few differentially expressed proteins. One of the functions of the PDL is to absorb the heavy occlusal forces of mastication with its tendon like collagenous structure (Mcculloch et al., 2000), by aligning collagen fiber bundles in the direction of forces and thereby increasing resistance to tensional forces. Tensional forces develop in the cervical and apical regions whereas the subapical region receives compressive forces, which may explain the lack of differentially expressed proteins.

**TABLE 2:** Proteomic expression change between P28 (1 week after occlusion) and P18 (pre-occlusal eruption).

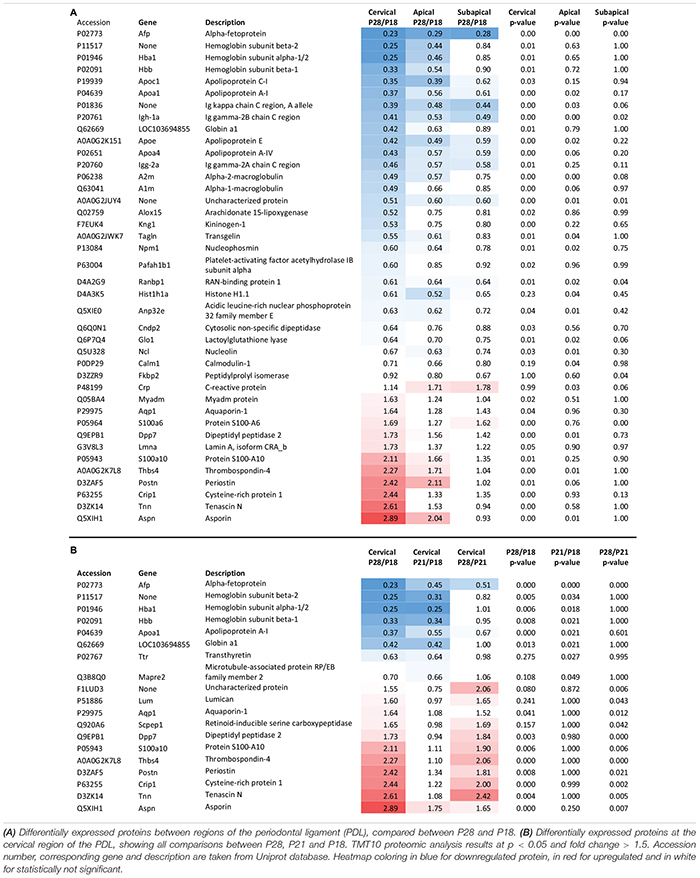

As the most changes were detected in the cervical region and the apical region seemed to mimic the same changes, we focused our analysis on the cervical PDL. Approximately half of the differentially expressed proteins in the cervical PDL increased gradually and showed statistically significant differences only between P28 and P18. The other half showed significant differences either between P21/P18 or P28/P21 (Table 2B). Interestingly, all the upregulated proteins had a late expression increase, with differences detected only between P28 and P21, while all the downregulated proteins showed differences already at P21, indicating that if triggered by occlusal contact the downregulation occurred in less than 24 h.

### Downregulation of Pro-metabolic Proteins Denotes PDL Maturation

**FIGURE 2:**
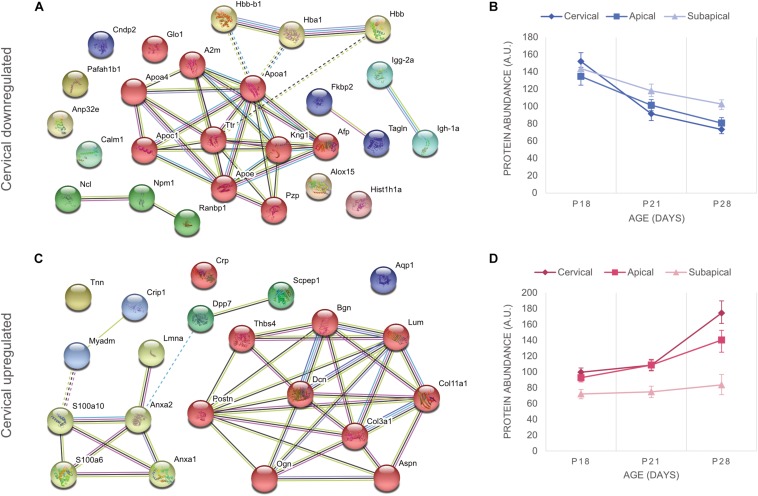
Differentially expressed proteins of the cervical PDL between ages P18 and P28 at fold change > 2 and *p*-value < 0.05. **(A)** STRING analysis revealed that downregulated proteins of the cervical PDL belong to two major groups of proteins, cholesterol and oxygen transporter proteins. https://version-11-0.string-db.org/cgi/network.pl?taskId=dVYaMgdf3BQb. **(B)** Graph of downregulated proteins at three ages and three PDL regions. All regions show progressive decrease of expression between P18 and P28. **(C)** STRING analysis shows two groups of proteins, matrisomal (red cluster) and matrisome-associated (yellow cluster). https://version-11-0.string-db.org/cgi/network.pl?taskId=MOt9XxpFbTAH. **(D)** Graph of upregulated proteins at three ages and three PDL regions shows upregulation only in cervical and apical proteins, essentially after occlusion between P21 and P28. Error bars represent confidence interval at 95%.

The downregulated proteins were uploaded to STRING to construct protein-to-protein interaction (PPI) networks. The majority of these proteins are implicated in the lipid metabolism, in particular to cholesterol transport (Figure 2A). Apolipoproteins are widely represented in the downregulated portion. Their main role is binding of lipids to form lipoproteins that are water-soluble particles enabling transport in the blood stream (Rubinsztein, 1995). They also interact with low density lipoprotein (LDL) receptors to facilitate lipid uptake and use by tissues for energy production (Mahley, 1988), as has been shown for LDL internalization by apolipoprotein E (ApoE) in fibroblasts (Innerarity et al., 1983; Datta et al., 2000). On the contrary, the ApoC1 and ApoA1 bind HDL molecules and enable the efflux of lipids from within cells. Transthyretin (Ttr) binds and transports thyroxine (Munro et al., 1989), which is a thyroid hormone known to increase metabolic rate (John-Alder, 1983; Johnstone et al., 2005). Decrease of lipid associated proteins indicate that cells decrease their metabolic rates which is consistent with cell maturation. Alpha-fetoprotein (Afp), a well-known stem cell marker (Kuhlmann and Peschke, 2006), was observed to be downregulated in all regions of the PDL, indicating that a general process of cell fate determination and cell maturation took place. Furthermore, we also observed decrease of hemoglobins, hemoglobin subunit alpha-1/2 (Hba1), hemoglobin subunit beta-1 (Hbb) and hemoglobin subunit beta-2-like (Hbb-b1), which are involved in oxygen binding and transport (Perutz, 1979; Bolognesi et al., 1997) and have been shown to decrease during cell maturation (Glowacki and Millette, 1965). Downregulation of these proteins takes place throughout the three regions and for the cervical region, most of the downregulation occurs between P18 and P21 (Figure 2B). This evidence indicates that upon establishment of occlusion a general process of tissue maturation takes place in the cervical and apical regions of the PDL.

### Core-Matrisomal and Matrisome Associated Proteins Are Upregulated During PDL Maturation

Protein-to-protein interaction networks from STRING analysis of upregulated proteins consisted of 21 nodes (Figure 2C). They revealed ECM proteins that have been classified by the Matrisome Project (Naba et al., 2016) into 10 core matrisome proteins (red cluster) and 4 matrisome-associated proteins (yellow cluster). The core-matrisome proteins consisted of 2 collagens (collagen α-1 type III, collagen α-1 type XI), 3 glycoproteins (periostin, tenascin-N, thrombonspondin-4) and 5 proteoglycans (asporin, biglycan, decorin, lumican, osteoglycin). The matrisome-associated proteins were 2 ECM-affiliated (annexin A1, annexin A2) and 2 secreted factors (S100-A6 and s100-A10 proteins) and were linked to lamin A, myeloid-associated differentiation marker and cysteine-rich protein 1, which are nucleus, membrane and cytoplasm proteins respectively. Annexins and S100 proteins are also known to have intracellular functions and their PPI network link with other intracellular functioning proteins indicates that even though they are members of the matrisome database, their localization in the PDL is not necessarily in the ECM. The upregulated proteins show a tendency to late expression following occlusal contact, as the curves increase mainly after P21 (Figure 2D). A clear gradient of upregulation can be observed between cervical and apical, whereas the subapical region shows no change. Upregulation following occlusal contact shows late expression of matrisomal proteins in the cervical and apical regions.

### Periostin Is Expressed on the Surface of Collagen α-1 (III)

**FIGURE 3:**
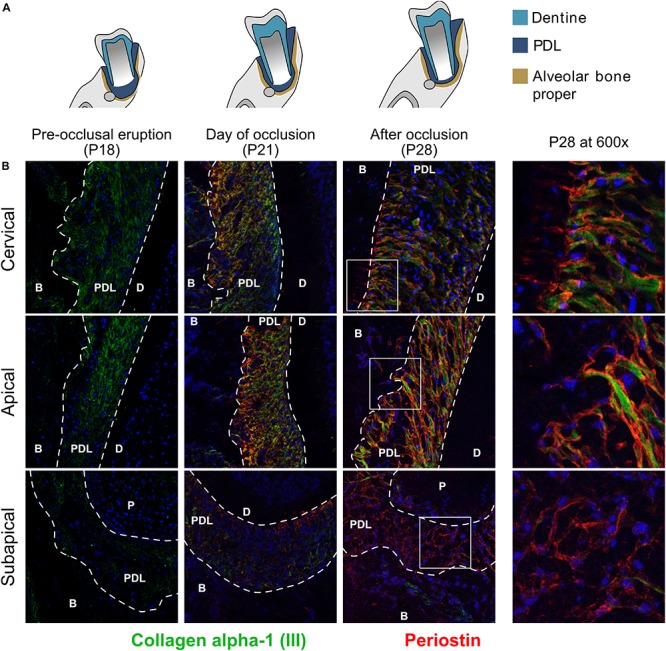
**(A)** Schematic representation of time points studied. **(B)** Collagen α-1 type III and glycoprotein periostin of the core-matrisome at three time points and three regions. Co-stained with collagen α-1 (III) (green) and periostin (red). Collagen α-1 (III) is expressed in the cervical and apical regions and undergoes rearrangement after occlusion. Periostin is only expressed after occlusion and in the three regions of the periodontal ligament. periodontal ligament (PDL), dentine (D), alveolar bone (B).

We co-stained two core matrisomal proteins, collagen α-1 type III (Col3a1) and periostin (Postn) (Figure 3). At P18 collagen α-1 (III) is present in the cervical and apical regions but doesn’t have any specific organization. Upon occlusion, we observed the appearance of organized collagen networks linking bone and cementum and at P28 the network shows distinct directionality arising from occlusal forces (Bernick, 1960). The subapical region showed no collagen type III expression. Periostin is expressed only after established occlusion and is more predominant in the cervical and apical regions, although it was also detected at P28 in the subapical region. Periostin was located on the surface of collagen fiber bundles and can be seen protruding into the alveolar bone (P28 cervical).

### Proteoglycans in the Periodontal Ligament Are Expressed in Specific Areas

**FIGURE 4:**
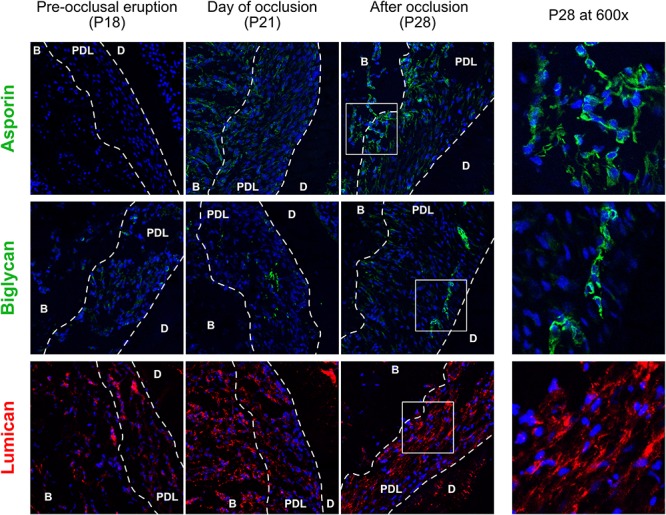
Core-matrisomal proteoglycans of the cervical periodontal ligament at three time points, staining of asporin (green), biglycan (green), and lumican (red). periodontal ligament (PDL), dentine (D), alveolar bone (B).

Staining of proteoglycans of the core-matrisome revealed that although they are all upregulated, their distribution within the PDL is not the same (Figure 4). Asporin was expressed primarily on the half of the PDL facing the alveolar bone in contrast to biglycan which was observed in cells near the cementum, while lumican was expressed ubiquitously. Asporin was not expressed at P18, increased slightly at P21 and was strongly expressed at P28. Biglycan is most probably localized inside cementoblasts at P28, meaning that these cells may produce cementum that contains biglycan. It is also interesting to remark that at P21 biglycan staining was found in fibroblasts in the center of the PDL space. It suggests that occlusal forces induced the differentiation of fibroblasts to cementoblasts capable of producing biglycan. Both asporin and biglycan show a perinuclear and cytoplasmic localization. Lumican expression was observed earlier than asporin and biglycan, already present at P18 and increased over time. At P21 expression is strong in the alveolar bone and P28 in the PDL.

### Matrisomal-Associated Proteins

**FIGURE 5:**
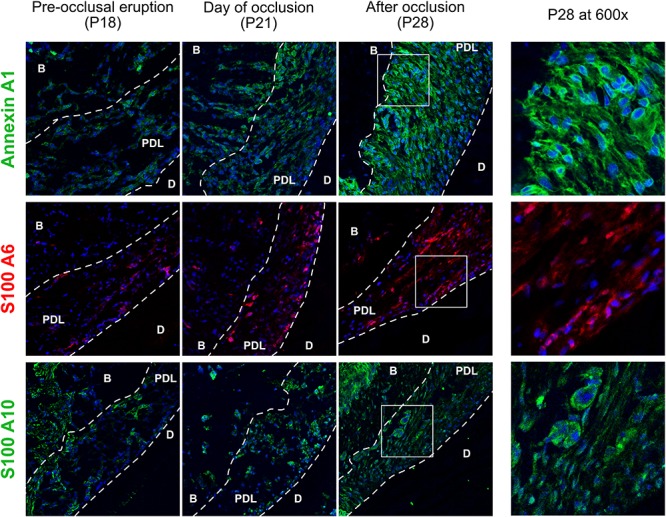
Matrisomal-associated proteins of the cervical periodontal ligament, stained for annexin A1 (green), S100-A6 (red) and s100-A10 (green). Periodontal ligament (PDL), dentin (D), alveolar bone (B).

Staining of three matrisomal-associated proteins show a differential expression pattern that confirmed the TMT10 proteomic results (Figure 5), all three proteins are gradually upregulated between P18 and P28. Annexin A1 is the most ubiquitous of the three and is perinuclear and cytoplasmic at P28. The S100 proteins expression is weaker than Annexin A1. S100-A6 is intranuclear at P21 in cementoblasts and a few osteoblasts. This finding is supported by Bozic et al. (2012) who observed S100-A6 protein in cementoblasts. S100-A10 is ubiquitously expressed and some cells show strong staining around the nucleus.

## Discussion

The PDL is a fibrous connective tissue that links two hard tissues, the alveolar bone of the jaws and the teeth. Its serves several purposes such as masticatory force absorption, tooth movement and tooth eruption, all of which require a highly adaptive and resistant tissue. The PDL is made up of two major components, the PDL fibroblasts and the ECM, which interact in order to adapt to external forces and ensure tissue homeostasis. We investigated the composition of the PDL with a proteome wide TMT10 profiling in three regions, the cervical, apical and subapical and used the matrisome project database to characterize the ECM of the PDL. The core-matrisomal protein group was increased, mostly due to a 2–3-fold higher ratio of collagens and proteoglycans. We identified the presence of collagens type II, IV, XI, XIV, XVI previously undescribed in the PDL. Collagen type IV has been shown to be slightly positive in PDL fibroblast of the dental follicle (Andujar et al., 1985) and to stimulate PDL fibroblast attachment (Giannopoulou and Cimasoni, 1996), but it hasn’t been identified as part of the mature PDL. The cervical and apical regions showed similar expression and we were able to identify 15 proteins characterizing these regions, which were for the most part matrisomal proteins. The only protein found to be differentially expressed between cervical and apical regions was the S100-A4. Although it has many functions, both intracellular and extracellular, S100-A4 primarily interacts with cytoskeletal proteins such as tropomyosin and actin to promote cell migration (Donato et al., 2013). It has also been shown to promote smooth muscle cell motility and proliferation (Schneider et al., 2007), which taken together with its predominant localization in the cervical region suggests that it plays a particular role in the response to occlusal forces. The subapical region showed very different expression profile, however it must be kept in mind that there was a cervical to apical gradient in maturation and the subapical region may not be mature at P28.

CAMs are transmembrane proteins that attach the cytoskeleton of the cell to the ECM components, of which the principal category that we identified were integrins. Integrins are heterodimeric transmembrane receptors composed of an alpha and beta subunit, to allow cell-to-matrix anchorage and mechanosignaling (Humphries et al., 2006). We detected two isoforms of the alpha subunit, integrin alpha 11 (Itga11) and integrin alpha V (Itgav) and one isoform of the beta subunit, integrin beta 1. Integrin α11β1 has been identified in the PDL (Zeltz et al., 2014) and in human PDL fibroblasts as a major receptor for collagen I, necessary for contractile functionality (Barczyk et al., 2009: Barczyk M. et al., 2013). Knockout mice for this receptor showed incisor eruptional problems and disorganized PDL structure, partially due to decreased MMPs in incisor fibroblasts (Popova et al., 2007; Barczyk M.M. et al., 2013). It has also been shown to regulate fibroblast migration in conjunction with platelet derived growth factor PDGF on fibroblasts (Popova et al., 2004). Integrin αV subunit has mostly been linked with the PDL in conjunction with the β3 subunit and integrin αVβ3 attaches indirectly to collagen III via glycoproteins we detected (Wu et al., 1996; Schvartz et al., 1999; Palaiologou et al., 2001), including periostin (Postn) (Watanabe et al., 2012; Matsuzawa et al., 2015) and its homolog TGF-β-induced (Tgfbi) (Son et al., 2013). Integrin αVβ1 has been implicated in the activation of TGF-β signaling in fibroblasts and subsequent tissue fibrosis (Reed et al., 2015) but its role in the PDL has not been described. Cytoplasmic proteins such as vinculin, talin2, filamin and actinin act as intermediates between integrins and the actin cytoskeleton (Brakebusch and Fässler, 2003) and play various regulatory roles in the context of mechanical strain to ensure proper attachment of cells (Humphries et al., 2007), for example by modulating integrin β1 binding to ECM (Pinto et al., 2013). These cytoplasmic cell adhesion proteins may enable the adaptive nature of the PDL, although the most well-known mechanosensor proteins are paxillin and FAK (Hytönen and Wehrle-Haller, 2016). Thrombospondin-4 is a transmembrane CAM, which was the only upregulated CAMs apart from periostin and thus may have an important role. It has been detected in the PDL (Lee et al., 2013) but its function was mostly studied in the context of cancer, where it promotes cell proliferation and migration (Chen et al., 2019, p. 4).

Based on our previous study (Denes et al., 2018), we hypothesized that the rapid change in eruption velocity seen during the transition from pre-occlusal to functional eruption may be in response to the appearance of occlusal forces. Occlusal loading influences the PDL width (Denes et al., 2013), structure (Mcculloch et al., 2000), and recovery (Terespolsky et al., 2002; Mine et al., 2005), therefore we examined differential expression during this transition to functional eruption with its first masticatory forces. The changes that occur during the transition to the functional eruption appear gradually, earlier in the cervical than the apical region. This suggests that the occlusal forces do not distribute equally in the PDL and have a different effect in different regions, possibly due to either a stretching force (cervical and apical regions) or a compressive force (subapical region). We concentrated on the differential expression of the cervical region to describe maturational changes in matrisomal proteins during the transition to functional eruption.

Alpha-fetoprotein has been used to characterize cell differentiation. It was observed to be downregulated in all regions of the PDL, which confirms that a general process of cell maturation is taking place upon establishment of occlusal contact. Pro-metabolic apolipoproteins were also downregulated indicative of decreased oxygen use of the cells, an overall more quiescent state and less proliferative. Cell maturation is also supported by decrease of ribosomal proteins nucleolin (Ncl), Ran binding protein-1 (Ranp1) and nucleophosmin (Ncm1) and decrease of protease inhibitors, T-kininogen-1 (Kng1), Alpha-1-macroglobulin (Pzp or A1m) and Alpha-2-macroglobulin (A2m) as both groups of proteins are essential to protein synthesis. This is also supported by upregulation of annexin A1 which has anti-proliferative properties (Alldridge and Bryant, 2003) and the rearrangement of the collagen type III fibers, with a directionality to absorb occlusal forces. Taken together, these elements provide reasonably strong evidence to support that appearance of occlusal contacts coincides with PDL maturation. A group of hemoglobins, transporters of oxygen, where seen to decrease upon P21, the day of first occlusal contact. It is possible that mRNA transcription is extremely rapid, but it is also possible that these changes are triggered by a different stimulus than occlusal forces. However, we believe that it may be due to occlusal forces that reduce the space in the ECM and partially collapse blood vessels, therefore leading to decrease in vascularization. Further investigation is needed to confirm either one of these hypotheses.

Periostin has been shown to be an essential protein in PDL integrity (Tabata et al., 2014) and its absence has been linked with periodontal disease (Rios et al., 2005). Its primary role is cross-linking of collagen fibers and thus participates in collagen fibrillogenesis (Romanos et al., 2014). Furthermore, periostin expression in response to occlusal forces appears necessary for maintaining a functional PDL (Rios et al., 2008) and decrease of occlusal forces by soft diet has been shown to partially rescue periostin−/− mice PDL (Rios et al., 2005, 2008). Periostin expression is also increased during orthodontic movement, where tension and compression appear in the PDL (Wilde et al., 2003). This is in line with our findings, which show that periostin expression in the PDL occurred after occlusal establishment. TGF-β signaling has been shown to mediate PDL cell response to occlusal forces by enhancing periostin production (Horiuchi et al., 1999) and blocking of TGF-β1 resulted in decrease of periostin production (Rios et al., 2008). Periostin co-localizes with collagen type III, suggesting that it may bind collagen type III and enhance cell-to-matrix adhesion via integrin αVβ3 and αVβ5 and promotes motility (Gillan et al., 2002). Although we did not detect integrin β3 or β5, it is possible that αVβ1 can also bind periostin. This evidence suggests that periostin plays a key role in establishing a structured and stable collagen matrix in the PDL, in response to occlusal forces, by enhancing the adhesion force of PDL fibroblasts to the ECM and ensuring the integrity of the PDL under tension. It is possible that this is the extracellular equivalent of the actin anchoring reinforcement under tension by the talin-vinculin complex (Ciobanasu et al., 2014).

Proteoglycans have been found to have multiple functions, from cell signaling and regulation of cell differentiation to mechanical properties such as resilience of the ECM to compressive forces (Kurylo et al., 2016). This mechanical property is called thixotropy and has been attributed to proteoglycans in skin (Finlay, 1978). Asporin regulates PDL fibroblasts (Kajikawa et al., 2014) via BMP-2 and PDL osteoclasts via receptor activator of nuclear factor κB ligand (RANKL) and *o*steoprotegerin (OPG) (Yu et al., 2018) as well as negative regulation of TGF-β (Kizawa et al., 2005) and bone morphogenetic protein 2 (BMP-2) (Yamada et al., 2008) and enhancement of fibroblast growth factor 2 (FGF-2) (Awata et al., 2015). Asporin’s regulation of osteogenic growth factors is in line with our results showing localization near the alveolar bone side of the PDL and in the bone itself. Given its control also of fibroblast differentiation, it is possible that asporin controls the interaction between PDL fibroblasts, osteoblasts and osteoclasts in the formation of Sharpey’s fibers. These observations are also supported by previous studies showing that induction of the RANKL/OPG system in PDL fibroblasts leads to osteaoclast genesis and bone resorption in the event of tooth movement and compressive forces in the PDL (Yang et al., 2018). This is a mechano-sensitive process (Jacobs et al., 2013). PDL fibroblasts have been shown to respond to mechanical strain by activation of molecules like Piezo-1 (Jin et al., 2014), β-2 adrenergic receptor (Adrb2) (Cao et al., 2014), prostaglandin (PG) E2/cyclooxygenase (COX)-2 (Römer et al., 2013) and Notch (Kikuta et al., 2015; Denes et al., 2019). Thus similarly to forces occurring during orthodontic tooth movement, masticatory forces seem to produce an osteogenic response in the PDL, possibly to remodel the alveolar socket shape or to reorganize Sharpey’s fibers. Also it is interesting to note that asporin has been shown to induce epithelial-mesenchymal transformation and it may have the same effect on the differentiation of PDL cells (Wang et al., 2017). The dental follicle cells are of epithelial origin, thus epithelial-mesenchymal transformation is consistent with differentiation of osteoblast progenitors. Furthermore, the absence of asporin has been linked to periodontal disease, such as periodontitis (Yamaba et al., 2015), which suggest a function in maintenance of periodontal integrity and health. Mechanical stimuli is known to induce osteogenic differentiation of PDL stem cells which most probably contributes to the maintenance of periodontal integrity (Zhang et al., 2012). However, in the case of periodontitis, PDL stem cells show decreased proliferation, increased osteoclastgenesis and inflammatory response (Liu et al., 2017).

Bigylcan shows the opposite tendency to aspirin, with localization near the cementum. This is in agreement with previous studies (Kurylo et al., 2016), which have shown biglycan in the cementum. The biglycan positive cells at P21 appear to be in the middle of the PDL space and may migrate to the cementum surface, as seen at P28. Furthermore, P28 shows a cluster of biglycan positive cells which may be Epithelial Rest Cells of Mallassez (ERM) that would then undergo epithelial-mesenchymal transformation. This is in line with a previous study on periodontal regeneration that demonstrated that connective tissue growth factor (CTGF), and partly FGF-2, induced production of collagen type III, biglycan and periostin in the PDL and significantly enhanced production of a mature PDL-like tissue (Dangaria et al., 2009). This underlines the importance of these proteoglycans and glycoproteins in the collagenous matrix. The role of lumican has yet to be identified, but it appears to be a more general proteoglycan as it can be seen on the entire width of the PDL and inside the alveolar bone.

Annexin A1 shows a strong increase between P18 and P28 and is omnipresent at P28, except for the line of cementoblasts where the staining is weaker. This seems to indicate that the majority of cells in the PDL decrease in proliferative activity upon transition to the functional phase of eruption, since annexin A1 has anti-proliferative function through activation of the ERK/MAPK pathway (Alldridge and Bryant, 2003) and has been shown to be upregulated in mesenchymal stem cells undergoing differentiation (Sun et al., 2006). The S100-A6 protein is much scarcer and appears to be secreted into the ECM. It is thought to be involved in cell proliferation and cytoskeletal dynamics and has been shown to be abundant in fibroblasts (Leśniak et al., 2009). It is well localized to the PDL, the alveolar bone doesn’t show much staining, contrary to the S100-A10 which can be seen in the alveolar bone and PDL and is localized peri-nuclear. The S100-A10 is known to interact with annexin A2 to assist trafficking of membrane proteins to the cellular membrane. Among these membrane proteins are the small GTPase of the Rho family, actin binding protein AHNAK and Cdc42 small effector protein 1, all of them implicated in the regulation of the actin cytoskeleton (Donato et al., 2013). Rho kinase pathway is also known to regulate the ECM composition (Yamamoto et al., 2018). This hints at a significant role of S100-A10 in organization of the ECM and adaptation of cell form for a functional PDL.

## Conclusion

The establishment of occlusal contact coincides with maturation of the PDL, defined by metabolic decrease, reduction of stem cell marker alpha-fetoprotein and increased organization of collagen type III fibers with directionality corresponding to occlusal forces. PDL maturation was accompanied by upregulation of matrisomal proteins, proteoglycans, glycoproteins, and matrisome-affiliated proteins that most likely promote the maturation process.

## Data Availability Statement

The datasets generated for this study can be found in the PRIDE PXD013379.

## Ethics Statement

The animal study was reviewed and approved by the Office Fédéral de la Sécurité Alimentaire et des Affaires Vétérinaire (OSAV) under authorization number GE/72/15.

## Author Contributions

BD performed the research, analyzed the data, and wrote the manuscript. AA-L performed the research, analyzed the data, and revised the manuscript. BW-H contributed reagents, revised the manuscript, and designed the research. SK designed the research and revised the manuscript.

## Conflict of Interest

The authors declare that the research was conducted in the absence of any commercial or financial relationships that could be construed as a potential conflict of interest.

## Footnotes

1 http://pepchem.org:35088/

2 www.string-db.org

## Supplementary Material

FIGURE S1 Experimental design. Four different tissue regions (cervical, apical, and subapical PDL and bone) from four biological replicates (rats) at three different time points (P18, P21, P28). Bone samples were not used in this study.

FIGURE S2 Design of the LCM samples labeling with TMT10plex reagents. Six TMT10 experiments were created. Groups 1–3 (G1–G3) refer to the age groups as shown in Supplementary Figure S1 and the M label corresponds to each individual rat. Rows correspond to TMT10 lanes and columns to different tags of the TMT Reagents Kit (TMT10plex Isobaric Mass Tag Kit).
