# Supplementary figures and images for: Core Matrisome Protein Signature During Periodontal Ligament Maturation From Pre-occlusal Eruption to Occlusal Function
Source: Front Physiol. 2020 Mar 5;11:174. doi: 10.3389/fphys.2020.00174 (PMC7066325; doi:10.3389/fphys.2020.00174)

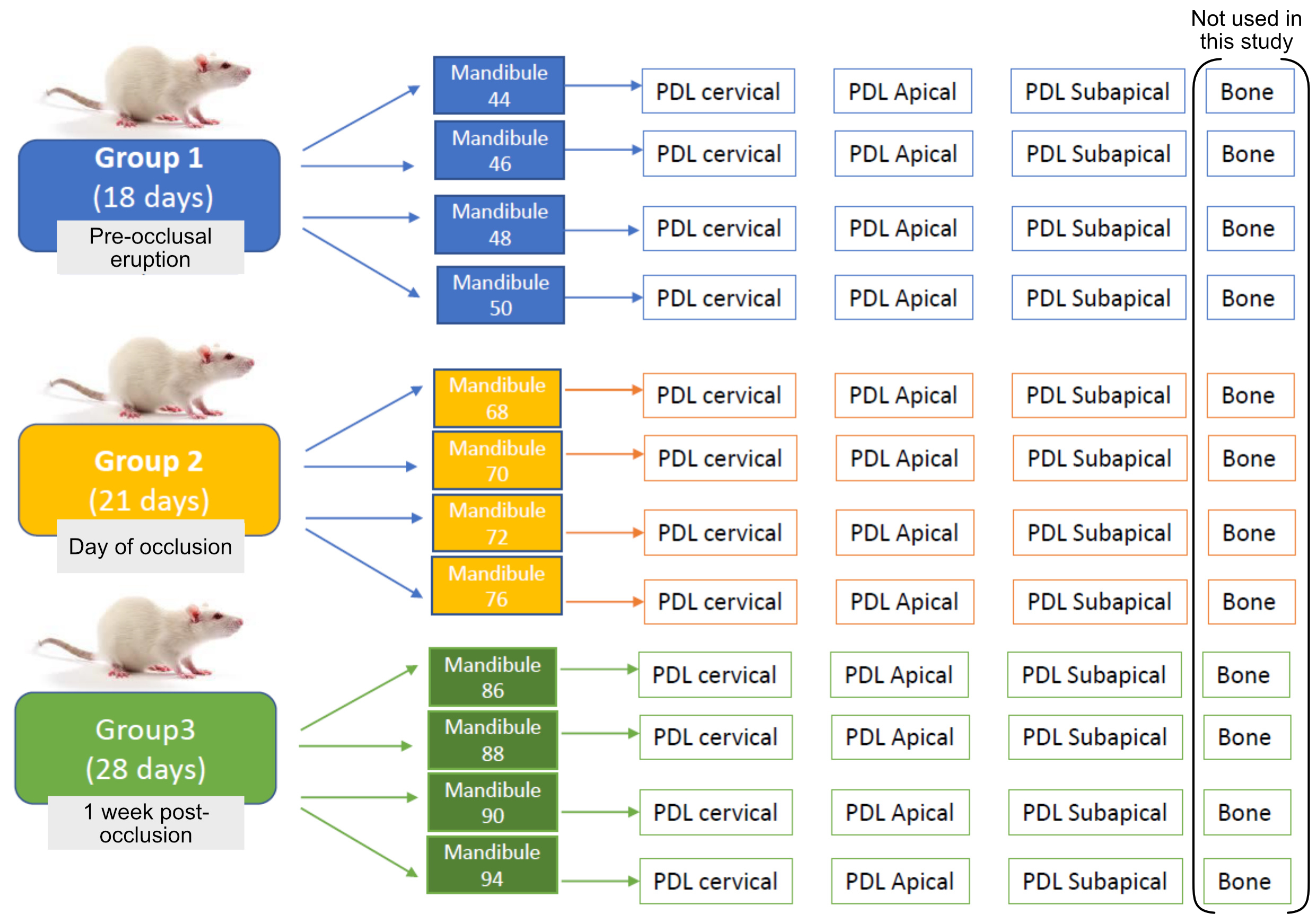

Supplement: FIGURE S1 — Experimental design. Four different tissue regions (cervical, apical, and subapical PDL and bone) from four biological replicates (rats) at three different time points (P18, P21, P28). Bone samples were not used in this study. [file Image_1.jpeg]

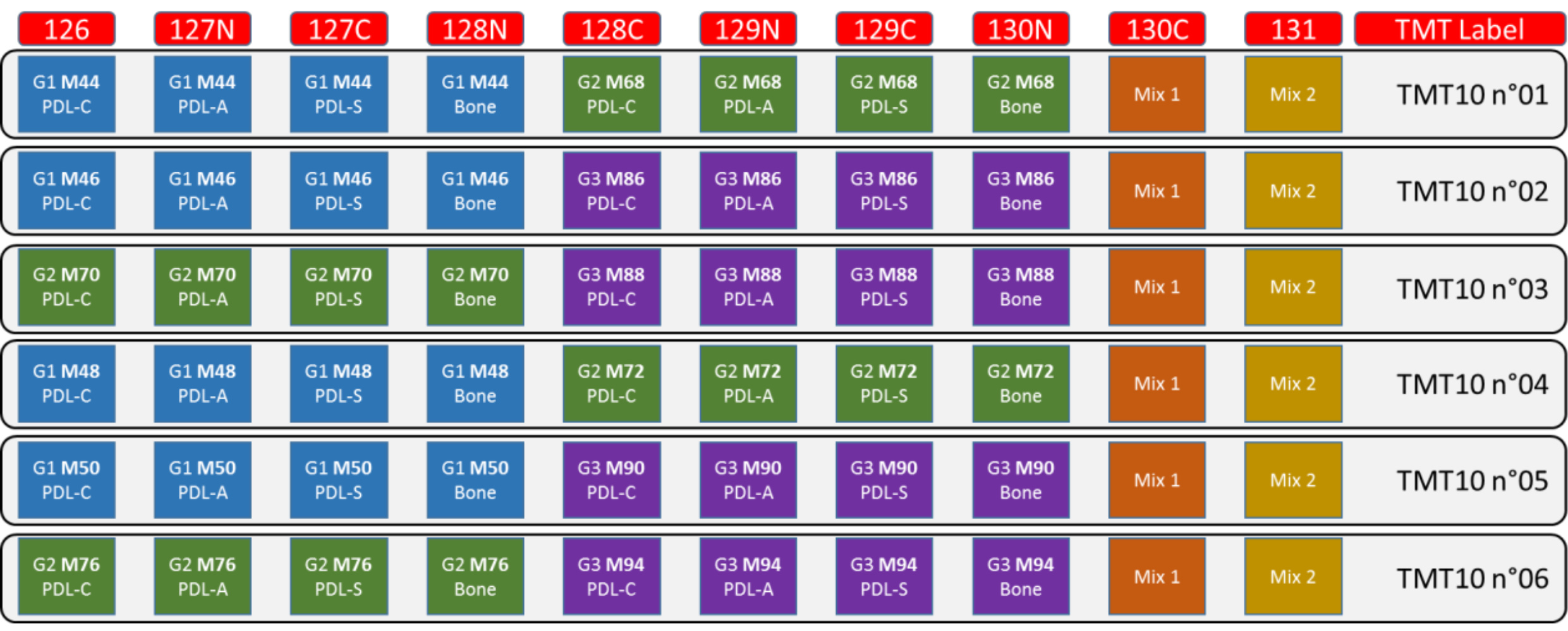

Supplement: FIGURE S2 — Design of the LCM samples labeling with TMT10plex reagents. Six TMT10 experiments were created. Groups 1–3 (G1–G3) refer to the age groups as shown in Supplementary Figure S1 and the M label corresponds to each individual rat. Rows correspond to TMT10 lanes and columns to different tags of the TMT Reagents Kit (TMT10plex Isobaric Mass Tag Kit). [file Image_2.jpeg]
